# Open fractures

**DOI:** 10.4103/0019-5413.43370

**Published:** 2008

**Authors:** Sudhir Babhulkar, HKT Raza

**Affiliations:** *Sushrut Hospital, Research Centre and Postgraduate Institute of Orthopaedics, Nagpur, India*; 1 *NSCB Medical College, Jabalpur, MP, India*

Open fractures (Gustilo I-III)^1^ continues to be a common injury with a high risk of complications such as wound infection and problems with healing of bone and soft tissues. The basic objectives in the management of open fractures are to prevent infection, reconstruct soft tissue defects and achieve bony union. With the availability of broad-spectrum antibiotics, antibiotic impregnated polymethylmethacrylate beads, pulse lavage and a choice of improved fracture stabilization and proficiency in plastic surgery procedures, the outcome of these injuries has improved.

The complications in open injuries during the course of fracture treatment dictate the use of methods believed to reduce the risk of complications, including urgent or emergent treatment and thorough debridement of wound, consisting of removal of all foreign materials, removal of devascularised tissues and reduction of the bacterial load introduced by disruption of the soft tissue envelope. Irrigation of the open fracture wound by sterile copius normal saline with or without additives with the combined use of systemic antibiotics and an antibiotic bead pouch for grade III B and III C fractures^2^ is important in removing/killing bacteria to optimize the wound healing. However, because of doubtful efficacy and potential toxicity, antiseptic irrigation should not be routinely employed. Pulsed lavage is instrumental in removing contaminants from wounds and reduces the bacteria as well as the wound inflammation and debris. The higher pressure settings (70 psi) have detrimental effects on bone healing, whereas low- to moderate pressure settings (15–25 psi) appear to balance the potential bone damaging effects with the proven contaminant clearing properties.

It is difficult to predict a subsequent infecting pathogen on the basis of initial wound cultures. Only 18% of the infections were caused by the same organism initially isolated in the perioperative cultures. Early wound coverage can prevent the emergence of hospital-acquired bacterial infection.^3^

Soft tissue closure depends on the attending surgeon's appraisal of the wound after debridement and bony fixation. Immediate primary closure, secondary closure and early flap coverage with or without the microsurgical reconstruction techniques can be used.^4^ The results showed that coverage within the first 72 h after injury provided superior results, with earlier bone healing and decreased rates of infection. In addition, the average length of hospital stay was notably diminished for the early flap against the delayed flap coverage. Delaying definitive reconstruction resulted in extensive fibrosis, which complicated the microvascular anastomoses and, in many instances, led to an additional loss of soft tissue and bone. In the “fix and flap” approach for grade III B and III C injuries of the tibia, the fractures were treated with immediate meticulous wound debridement with lavage, skeletal stabilization and definitive soft tissue coverage with a vascularized muscle flap and split-thickness skin graft.^4^ The goal was to obtain coverage within 72 h of the injury. The deep infection rate was 6% for patients with early flaps and 30% for those with late flaps.^5^ The flap failure rate was 3.5%. The authors suggested that delay in coverage is not necessary if healthy soft tissue can be imported reliably into the zone of injury.^4^,^5^ Despite studies supporting early closure, the major argument against primary wound closure is its association with the occurrence of gas gangrene. Obvious exceptions to immediate closure include wounds containing gross contamination with feces, dirt or stagnant water as well as farm-related injuries or freshwater boating accidents.

Various methods of fracture stabilization include markedly improved external fixators and intramedullary (IM) devices. Modern methods of early surgical fixation provide excellent stabilization of the injury zone, allow early joint range of motion and the possibility for immediate or early soft tissue cover by flaps. The use of plate, external fixator and IM nailing (reamed or unreamed) are under investigation and we are in the process of evolving consensus. There is evidence from a pooled analysis of randomized trials that reamed IM nailing of lower extremity long bone fractures significantly reduces the rate of non-union and implant failure in comparison with non-reamed nailing. Reamed nails, while destructive to the endosteal blood supply, afford greater stability at the fracture site due to their larger size and eliminate two-thirds of the non-union that occurs with non-reamed nailing. The unreamed nail insertion using smaller diameter nails shows that the rates of implant failure, delayed union, malunion and non-union seem to be higher than after reamed nail insertion. Secondary procedures such as exchange nailing and bone grafting appear to be needed more often to obtain union after unreamed tibial nail insertion. More recently, studies have revealed that up to 48% of open tibia fractures treated with small diameter IM nails inserted without reaming require a secondary procedure to achieve union and that there is a significant problem of interlocking screw failure with this technique. In addition, surgeons may be more comfortable allowing early weight bearing when stronger, better-fitting nails with larger interlocking bolts have been used for fixation. Keating et al. found no differences in union rates or infection rates between reamed and unreamed nail insertion with open fractures. They also reported that nails inserted after reaming had fewer implant failures than smaller diameter nails inserted without reaming.^6^–^8^

The major concern in connection with the nailing of severe open fractures of long bones is infection. The incidence of infection following reaming and nailing of open fractures of tibia is reported between 14 and 33%. Hence, in early days, external fixation was indicated as treatment of choice in type II and III open tibial fractures. But, as of today, after thorough debridement and irrigation, primary interlocked nailing after reaming is indicated in open fractures up to grade III A and B because external fixation alone, especially in unstable fractures, is associated with malunion, delayed union, loss of reduction, refracture, pin tract infection and non-union with the incidence ranging from 21 to 55%. However, in compound Gr III fractures with lots of contamination, the problems can be avoided by secondary IM nailing with a marked reduction in the rate of complications.^9^ The interval between removal of fixator and nailing may be an average of 3 weeks; meanwhile, the limb should be immobilized in a plaster slab. Furthermore, an increased rate of complications, especially infections, was not observed when nail insertion with reaming was performed. In animal models, the reaming process has been found to produce a paradoxical increase in the periosteal blood flow, so that the overall limb perfusion and fracture callus is not affected.

Advances in initial wound debridement and irrigation, access to broad-spectrum antibiotic coverage and experience with modern fracture stabilization techniques enable a more aggressive approach to be taken to open fracture management. The increasing incidence of resistant nosocomial infections and the cost implications of a dogmatic delayed-closure strategy wound care protocols for open fractures should be re-evaluated. The best clinical practice may be adoption of a treatment plan that allows for the earliest possible soft tissue coverage over a clean, stable, viable zone of injury. If these parameters are achieved at the time of initial debridement, primary wound closure appears to be a reasonable treatment option. An orthopaedic surgeon should proceed with aggressive debridement of all open wounds emergently. Although the “adequate debridement remains a difficult technical problem,” all non-viable tissue must be removed while as much functional tissue as possible is spared. Likewise, proper tissue-closure tension and optimal wound-closure technique are difficult to define but can be summarized as the methods that are not anticipated to cause additional tissue necrosis. When the possibility of progressive tissue necrosis is uncertain, the wound can be closed initially and subsequently opened, if necessary, for a second exploration.

Early soft tissue restoration has dramatically improved the outcome of these fractures. A better understanding of neurovascular supply and microsurgical techniques had led to reliable cover of traumatic musculoskeletal defects. Progressive refinement in the fixation of fractures and early bone grafting have reduced the fracture time to union. The dedicated team of orthopaedic surgeon and plastic surgeons with combined efforts to treat soft tissue injury and skeletal fixation of open fractures has further improved the outcome and reduced the morbidity.^5^ We now advocate the adequate debridement and skeletal stabilization by senior members of the team as an urgent procedure rather than poor emergency operation.

The scenario in developing countries is little different because such patients report late, beyond 24 h, without receiving proper first aid such as wound toilet, dressing and splintage and are stitched badly without proper wound toilet and debridement. The wounds are already infected with poly bacterial infections. Most of the hospitals in the periphery lack basic infrastructures hence state-of-the-art treatment as stated above cannot be offered. By the time they reach a tertiary care hospital, they already have compartment syndrome with necrosed skin and underlying muscles. They may or may not be given proper immobilization resulting in more soft tissue damage and tissue edema. They have pus-discharging fractures with deformed, extremely scarred, stiff proximal and distal joints. The bones are osteopenic with atrophic bone ends. It is a challenge to the clinical acumen of the surgeons.

For developed countries, the research to achieve well aligned, painless, normal extremity has progressed significantly; for developing countries, we need to devise the strategies to treat these difficult fractures in the available infrastructure to achieve a painless, mobile and well-aligned limb, particularly when they present late with infected, atrophic, osteopenic bone with scarred limb and stiff contractured joints. The treatment in such cases is prolonged and needs repeated hospitalization with an added element of uncertainty. It has lots of financial hardships and hence it is most important to develop an objective score to decide which limbs should be amputated at first instance.

The present symposium by the Indian Journal of Orthopaedics is an effort in this direction. It includes a review article on the utility of scores in the decision of salvage or amputation. The article by William W. Cross III and Swtontkowski reviews the “treatment principles in the management of open fractures” and another by Antino Rios-Luna discusses “pearls and tips in coverage of tibia after a high energy trauma.” Two original articles analyze the incidence of infection and other issues related with early and late IM nailing.

Besides treating such difficult clinical problems, there is a need to formulate the standard protocols of the management of open fractures that could be adopted at rural centers and definitive protocols of the management adopted at district hospitals, with limited resources.

## References

[1] Gustilo RB, Anderson JT (1976). Prevention of infection in complex trauma of one thousand and twenty five open fractures of long bones: Retrospective and prospective analysis. J Bone joint Surg Am.

[2] Keating JF, Blachut PA, O'Brien PJ, Meek RN, Broekhuyse H (1996). Reamed nailing of open tibial fractures: Does the antibiotic bead pouch reduce the deep infection rate. J Orthop Trauma.

[3] Bhandari M, Guyatt GH, Swiontkowski MF, Schemitsch EH (2001). Treatment of open fractures of the shaft of tibia: A systematic overview and meta-analysis. J Bone Joint Surg Br.

[4] Gopal S, Giannoudis PV, Murray A, Mathews SJ, Smith RM (2004). The functional outcome of severe open fractures managed with early fixation and flap coverage. J Bone Joint Surg Br.

[5] Naique SB, Pearse M, Nanchahal J (2006). Management of severe open tibial fractures: The need for combined orthopaedic and plastic surgical treatment in specialized centres. J Bone Joint Surg Br.

[6] Bhandari M, Guyatt GH, Tong D, Adili A, Shaughnessy SG (2000). Reamed versus nonreamed intramedullary nailing of lower extremity long bone fractures: A systematic overview and meta-analysis. J Orthop Trauma.

[7] Keating JF, O'Brien PJ, Blachut PA, Meek RN, Broekhuyse HM (1997). Locking intramedullary nailing with or without reaming for open fractures of tibial shaft. J Bone Joint Surg Am.

[8] Keating JF, Blachut PA, O'Brein PJ, Court-Brown CM (2000). reamed nailing of Gustilo grade IIB tibial fractures. J Bone Joint Surg Br.

[9] McGraw JM, Lim EV (1988). Treatment of open tibia-shaft fractures: External fixator and secondary intramedullary nailing. J Bone joint Surg Am.

